# Telecommuting-related health outcomes during the COVID-19 pandemic in South Korea: a national population-based cross-sectional study

**DOI:** 10.1186/s12889-023-15271-0

**Published:** 2023-03-23

**Authors:** Seung-Woo Ryoo, Jin-Young Min, Dong-Wook Lee, Baek-Yong Choi, Juho Choi, Ho-Yeon Kim, Kyoung-Bok Min

**Affiliations:** 1 Department of Preventive Medicine, College of Medicine, Seoul National University, 103 Daehak-ro, Jongno-gu, Seoul, 110-799 Republic of Korea; 2 Veterans Medical Research Institute, Veterans Health Service Medical Center, Seoul, Republic of Korea; 3 Integrated Major in Innovative Medical Science, Seoul National University Graduate School, Seoul, Republic of Korea

**Keywords:** Telecommuting, Occupational health COVID-19, Gender difference, National population survey

## Abstract

**Background:**

Telecommuting has expanded greatly during the COVID-19 pandemic. Since the advent of remote working from home, there has been an ongoing controversy about the positive or negative health-related impact of telecommuting. This study aimed to investigate change in the occupational health risk in South Korean workers involved in telecommuting during the pandemic period compared to daily commuters.

**Methods:**

A population-based cross-sectional study of South Korean workers using the secondary data from the 6th Korean Working Conditions Survey (2020–2021) was designed. A total of 12,354 white-collar wage employees were selected as the study sample. Telecommuting, depression, anxiety, insomnia, fatigue, musculoskeletal pain, headache-eye strain, absenteeism, and presenteeism were measured by self-reported data. Multiple logistic regression models, including gender stratification analysis, were used to estimate the adjusted odds ratio (AOR) with a 95% confidence interval (CI) for the health outcomes of telecommuters.

**Results:**

Among the study population, 338 males and 318 females were reported to be telecommuters. The entirely adjusted regression model showed a positive association between telecommuting and anxiety (AOR = 2.82; 95% CI, 1.93–4.10), insomnia (AOR = 1.93; 95% CI, 1.27–2.92), fatigue (AOR = 1.76; 95% CI, 1.30–2.37), musculoskeletal pain (AOR = 1,76; 95% CI, 1.33–2.32), headache-eye strain (AOR = 1.94; 95% CI, 1.48–2.54), presenteeism (AOR = 1.66; 95% CI, 1.20–2.28) respectively. Gender difference was identified in that only female telecommuters had a higher risk of depression (AOR = 1.62; 95% CI, 1.04–2.53) and insomnia (AOR = 2.07; 95% CI, 1.26–3.41) than daily commuters in the adjusted model.

**Conclusion:**

Telecommuting was significantly associated with an increased risk of various health problems among South Korean workers and females were identified as a more vulnerable group. Although further research is required to ascertain the causal relationship, public health intervention should be considered to prevent the negative effects of telecommuting.

**Supplementary Information:**

The online version contains supplementary material available at 10.1186/s12889-023-15271-0.

## Background

Telecommuting is a work arrangement in which workers perform all or parts of their duties away from the company’s physical location. Spatial separation between business and remote worksites and increased cooperation over national boundaries are considered attractive characteristics of telecommuting [1]. Advancements in technology for remote work have led to a gradual increase in the number of teleworkers across the labor market that persists to this day [2].

Several studies have focused on the health advantages and disadvantages of telecommuting. For example, a study showed that telecommuters had positive mental health-related outcomes, including reduced stress, enhanced work-life balance, and less time pressure and exhaustion than those working in the office [3]. In the contrary, an association between telecommuting and depression has been identified, although the relationship depends on the working days spent on telecommuting [4]. Other occupational mental disorders among telecommuters, such as anxiety and adjustment disorders, are yet to be studied. Telecommuters are also less likely to be exposed to traffic accidents and air pollution in urban areas, which can mitigate the cumulative risk of acute trauma and respiratory diseases [5, 6]. While telecommuters are vulnerable to physical illness due to poor workstation design or working outside of duty hours [5, 7]. Indeed, prior researchers have found a significant decrease in self-reported health and a high prevalence of musculoskeletal pain [3, 5]. Irregular work schedules induce sleep disorders and oculopathy in workers, especially at night work [8, 9]. However, further evidence of these outcomes is required. Other marginalized concerns of telecommuting are the risks of absenteeism and presenteeism [10]. Whether telecommuters are more likely to take sick leave (absenteeism) or continue working despite being sick (presenteeism) is closely related to their vocational life. Therefore, relevant epidemiological studies are needed.

The inconsistent hypotheses and findings from previous studies may be attributable to the mediating role of psychosocial working conditions in the relationship between telecommuting and workers’ health. At individual level, work-related stress or physical tiredness varies depending on a telecommuter’s extent of task autonomy, relationship with co-workers, and work-family conflict [11]. This may be a result of the blurred boundaries between work and family life accompanied by mental and physical stress [12, 13]. It is noteworthy that the negative aspects of telecommuting were found to be higher among females, who are mainly responsible for childcare and housework, while only male workers enhanced their quality of life and relieved stress through teleworking [14, 15].

The COVID-19 pandemic has led to a worldwide expansion in telecommuting [16, 17]. To contain the spread of the devastating respiratory virus, telecommuting has been encouraged globally in public sectors and industries to date. As in other countries, telecommuting has been actively implemented in South Korea since the beginning of the COVID-19 pandemic due to robust information and communications technology (ICT) infrastructure and aggressive social distancing policies [18]. Telecommuting is expected to spread worldwide even after the endemic phase of COVID-19 [19]. The unforeseen increase in the telecommuting population has made it necessary to investigate potential hazards to the health of telecommuters and establish effective countermeasures [5]. Hence, it is important now more than ever to compile epidemiological evidence on the health-related aspects of telecommuting and drafting adequate health policies. Previous studies have suggested the potential health risks associated with telecommuting, but most of them focused on a specific occupational group (i.e., information technology workers and financial employees) or were conducted without a homogeneous control group [3, 20]. Thus, a comparative study with a large, representative sample is required.

In the current study, we compared physical and mental health issues between telecommuters and daily commuters using a large representative Korean working population. The positive and negative aspects of telecommuting are influenced by gender, and gender gaps exist in terms of housework, work disruptions, supportive social policies, and infrastructure [14, 15, 21]. Therefore, we conducted a gender-stratified analysis to explore whether the association between telecommuting and health problems differed between male and female workers.

## Materials & methods

### Data source and study population

We conducted a population-based cross-sectional study among South Korean workers who actively labored during the pandemic, to investigate the association between telecommuting and various health-related outcomes. Data from the 6^th^ Korean Working Conditions Survey (KWCS) conducted by the Korea Occupational Safety and Health Research Institute were used. The KWCS produces cross-sectional survey data on various aspects of working conditions in South Korea to provide insights into occupational health promotion measures. Questionnaire items are based on the updated version of the European Working Conditions Survey (EWCS) [22]. A total of 50,538 workers aged ≥ 15 years in South Korea were selected to participate in the survey. The survey was held during the COVID-19 pandemic from 5, October 2020, to 12, December 2020 and from 27, January 2021 to 11, April 2021. The KWCS comprises public data with guaranteed confidentiality; therefore, this study was exempted from review by the Institutional Review Board of Seoul National University College of Medicine.

### Sampling design

**Fig. 1:**
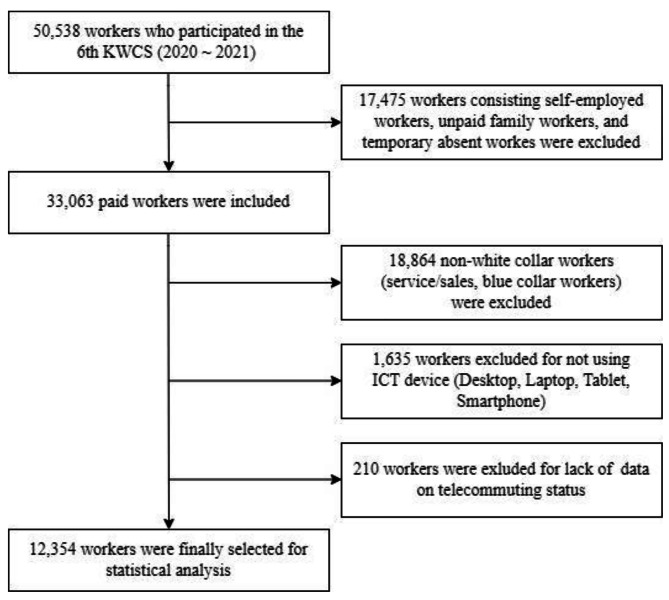
Sample selection with inclusion & exclusion criteria

Figure 1 shows the sample selection process. The following exclusion criteria were applied [23]: first, self-employed workers, unpaid family workers, and absent temporary workers (n = 17,475) were excluded, as telecommuters are workers paid by employers. In addition, workers with blue-collar or service/sales jobs (n = 18,864) and no ICT device usage (n = 1,635) were excluded because telecommuting requires an ICT-friendly environment to communicate with coworkers [23]. After further exclusion of participants who refused to answer the question on the exposure variable (n = 210), the final sample comprised 12,354 participants.

### Definition of variables

Telecommuting status was defined as the exposure variable. Telecommuters were identified by their responses to the following question regarding the workplace: “Over the past 12 months, how often have you worked at the following place? Response: D) my home.”. Aligned with the abovementioned definition of “telecommuting” which demands partial replacement of typical working hours, those who worked from home “occasionally” or more often were categorized as “telecommuters”.

Eight health-related indicators were defined as the outcome variables. Depression was measured using the World Health Organization Five Well-being Index, which is a widely acknowledged tool for screening depression risk with high sensitivity and specificity [24]. Responses for the five items are rated on a 6-point Likert scale, where the lower points on the scale indicate a higher score [for example, 1 = all the time (score 5) to ~ 6 = at no time (score 0)]. The sum of the scores multiplied by 4 ranged from 0 to 100. Based on a previous study, individuals with a score below 50 were coded as depression events [24]. The Minimal Insomnia Symptom Scale (MISS), composed of a 3-item sleep questionnaire, was used to measure the risk of insomnia. Responses for each item are rated on a 5-point Likert scale where the higher points on the scale indicate a higher score [for example, 1 = daily (score 0) to 5 = never (score 4)]. The sum yielded a maximum score of 12, and individuals who scored more than 6 were considered to have a high risk of insomnia [25]. Other illnesses were determined using questions regarding experiences with health problems over the past 12 months. Participants who responded “yes” to at least one of the three items regarding physical pain (low back, upper limbs, and lower limbs) were coded as musculoskeletal pain events. Correspondingly, those who responded “yes” to the other three items (headache/eye strain, anxiety, and fatigue) were considered affected individuals for each variable. Absenteeism was defined as the number of days absent due to health problems over the past 12 months. Individuals with more than one day of sick leave are considered to have a high risk of absenteeism. Presenteeism was defined by the question inquiring about working while ill over the past 12 months. Individuals that responded “yes” were considered to be prone to suffering from presenteeism.

Other variables of interest were measured as covariates. Sociodemographic characteristics were age (15–29, 30–39, 40–49, 50–59, or ≥ 60 years), gender (male or female), education level (below high school, college, or graduate school), monthly income (per 10,000 Korean Won; KRW: <200, 200–299, 300–399, or ≥ 400; 1 United States dollar is equal to 1,000 ~ 1,300 KRW), and household number (1, 2, or ≥ 3). Occupational characteristics included working hours (< 40, 40–52, or > 52 h), shift work, and type of occupation (manager, professional, or clerk). Working hours were categorized according to the Labor Standard Act of Korea. Since there were a limited number of telecommuters with managerial jobs in our sample, occupation type was classified into the following two groups: managers/professionals and clerks.

### Statistical analysis

A descriptive analysis was performed to assess the distribution of telecommuters’ sociodemographic and occupational characteristics. The chi-square and Fisher’s exact tests were used to evaluate the differences in characteristics among the study groups. We conducted multiple logistic regression analyses to estimate the adjusted odds ratio (AOR) with 95% confidence intervals (CI) and to verify associations between telecommuting and health-related outcomes. Moreover, a gender-stratified analysis was conducted to investigate the differences in the effects of telecommuting on health between males and females. Both the unadjusted and adjusted models were used for each regression analysis. Model 1 was adjusted for sociodemographic characteristics, and Model 2 was further adjusted for occupational characteristics. All the analyses were conducted using the survey weight values assigned to each participant provided by the 6th KWCS to obtain unbiased estimators for the parameters. The final weight was calculated by multiplying the design, non-response, and post-stratification weights. As a consequence, the structure of the entire population and survey participants were matched in terms of age, gender, region, occupation type, and employment status. The analyses were conducted using SAS software (version 9.4; SAS Institute, Cary, NC, USA), and statistical significance was set at a P value of 0.05 or less.

## Results

Table 1 shows the sociodemographic and occupational characteristics of the study population according to telecommuting status. A descriptive analysis including weighted survey values resulted in an augmented sample size (n = 15,451) compared with the number of actual participants (n = 12,354). Among the study participants, 338 were males and 312 were females telecommuters. Compared with daily commuters, male telecommuters were older (≥ 40 years, 64.02%), whereas female telecommuters were younger (15–39 years, 58.02%). Both male and female telecommuters had higher educational levels, whereas a low proportion of daily commuters attended graduate school (7.40%). Notably, male telecommuters earned the highest income (≥ 4 million KRW/month, 54.16%), and female telecommuters worked for the shortest duration (< 40 h/week, 27.77%) among the three groups. There was no significant difference in the number of households according to telecommuting status. A lower proportion of telecommuters engaged in shift work and a higher proportion were classified as managers/professionals by occupation type.

**Table 1:** Sociodemographic and occupational characteristics of the participants according to telecommuting status

		Telecommuter				Daily commuter			
Characteristics		Male		Female				p-value	
Total		338		312		14,801			
Age									
	15–29	21	(6.24)	71	(22.68)	2,597	(17.54)	0.0002	
	30–39	101	(29.74)	110	(35.34)	4,656	(17.54)		
	40–49	130	(38.35)	85	(27.23)	4,490	(31.46)		
	50–59	63	(18.49)	43	(13.86)	2,575	(31.46)		
	≥ 60	24	(7.19)	3	(0.89)	483	(30.34)		
Education									
	Below high school	19	(5.68)	33	(10.47)	1,444	(9.76)	< 0.0001	
	College	248	(73.77)	232	(74.44)	12,259	(82.85)		
	Graduate school	69	(20.54)	47	(15.10)	1,094	(7.40)		
Income (10,000KRW/month)									
	< 200	13	(4.28)	75	(25.96)	1,624	(11.59)	< 0.0001	
	200–299	49	(15.59)	114	(39.55)	4,698	(33.50)		
	300–399	81	(25.96)	51	(17.53)	3,721	(26.54)		
	≥ 400	169	(54.16)	49	(16.96)	3,978	(28.37)		
Household number									
	1	23	(6.91)	28	(9.00)	1,075	(7.26)	0.9043	
	2	47	(13.91)	44	(14.10)	2,082	(14.07)		
	≥ 3	268	(79.18)	240	(76.90)	11,645	(78.67)		
Working hours (/week)									
	< 40	34	(10.03)	87	(27.77)	1,040	(7.03)	< 0.0001	
	40–52	297	(87.73)	220	(70.51)	13,419	(90.66)		
	> 52	8	(2.24)	5	(1.73)	342	(2.31)		
Shiftwork									
	No	334	(98.67)	304	(99.05)	14,142	(95.81)	0.02	
	Yes	5	(1.33)	3	(0.95)	619	(4.19)		
Occupation Type									
	Manager /Professional	235	(69.53)	190	(60.78)	7,429	(50.19)	< 0.0001	
	Clerk	103	(30.47)	122	(39.22)	7,372	(49.81)		

Values are presented as number (%)

**Fig. 2:**
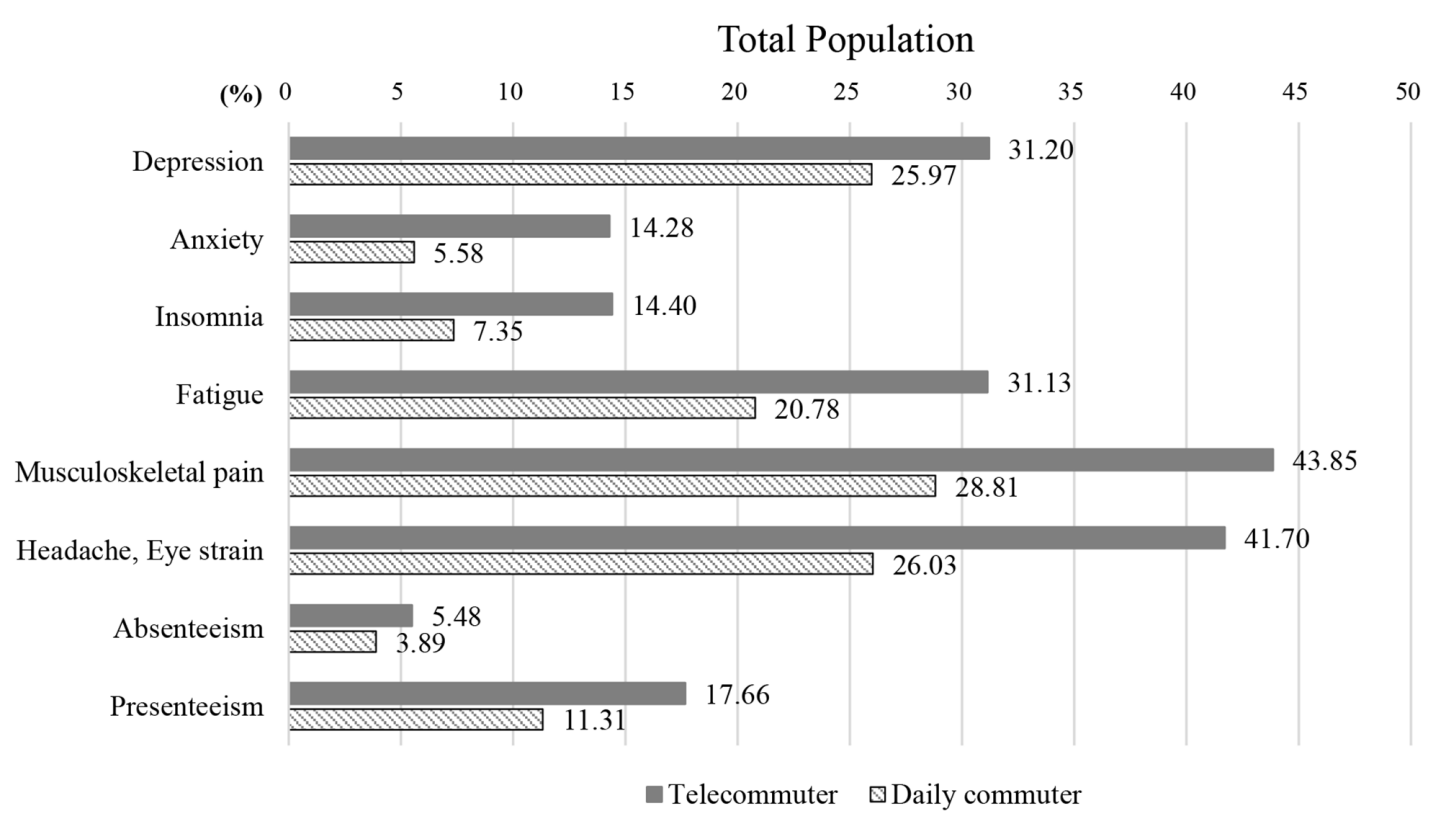
Percentage of affected participants in each health outcome according to telecommuting status

The associations between telecommuting and various health-related outcomes in the total population are shown in Table 2. Figure 2 shows a comparison of the number and percentage of participants affected by each outcome according to telecommuting status. In terms of mental health, telecommuters were more likely to report anxiety (AOR = 2.82; 95% CI, 1.93–4.10) and insomnia (AOR = 1.93; 95% CI, 1.27–02.92) than daily commuters after adjusting for all potential covariates. However, no statistically significant association was observed for depression in both the unadjusted (OR = 1.29; 95% CI, 0.96–1.74) and fully adjusted models (AOR = 1.34; 95% CI, 0.98–1.84). For all physical illness-related outcomes, telecommuters were consistently at a higher risk of suffering from health problems than daily commuters. After adjusting for sociodemographic and occupational factors, telecommuting was significantly associated with fatigue (AOR = 1.76; 95% CI, 1.30–2.37), musculoskeletal pain (AOR = 1.76; 95% CI, 1.33–2.32) and headache/eye strain (AOR = 1.94; 95% CI, 1.48–2.54). While absenteeism was not associated with telecommuting (adjusted model 2: AOR = 1.37; 95% CI, 0.84–2.24), a significant association was observed between presenteeism and telecommuting (adjusted model 2: AOR = 1.66; 95% CI, 1.20–2.28).

**Table 2:** Odds ratios with 95% CIs for health-related outcomes of telecommuters

	N (%)^a^		Unadjusted Model	Adjusted Model 1^b^	Adjusted Model 2^c^
Depression	200 (31.20)		1.29 (0.96–1.74)	1.31 (0.96–1.80)	1.34 (0.98–1.84)
Anxiety	93 (14.28)		2.82 (1.97–4.04)^*^	2.67 (1.83–3.89)^*^	2.82 (1.93–4.10)^*^
Insomnia	93 (14.40)		2.12 (1.47–3.06)^*^	1.89 (1.25–2.84)^*^	1.93 (1.27–2.92)^*^
Fatigue	202 (31.13)		1.72 (1.29–2.30)^*^	1.72 (1.28–2.31)^*^	1.76 (1.30–2.37)^*^
Musculoskeletal pain	284 (43.85)		1.93 (1.50–2.48)^*^	1.77 (1.35–2.33)^*^	1.76 (1.33–2.32)^*^
Headache, Eye strain	270 (41.70)		2.03 (1.59–2.61)^*^	1.96 (1.50–2.56)^*^	1.94 (1.48–2.54)^*^
Absenteeism	36 (5.48)		1.44 (0.90–2.28)	1.38 (0.85–2.25)	1.37 (0.84–2.24)
Presenteeism	115 (17.66)		1.68 (1.24–2.28)^*^	1.64 (1.20–2.25)^*^	1.66 (1.20–2.28)^*^

CI: confidence interval

^a^Number and percentage of telecommuters affected in each outcome

^b^Adjusted for gender, age, education, income, household size

^c^Adjusted for gender, age, education, income, household size, working hour, shift work, occupation type

^*^P-value < 0.05

**Fig. 3:**
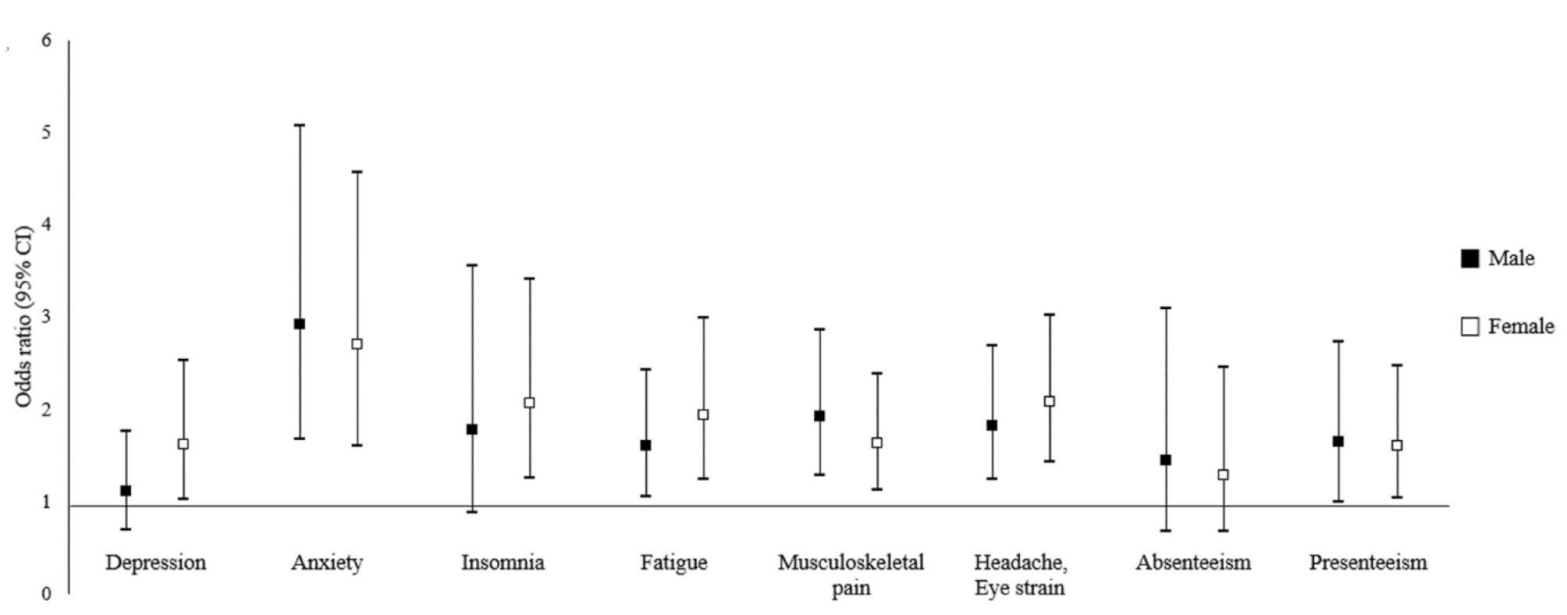
Adjusted odds ratios with 95% confidence intervals for health problems of telecommuters by gender. Filled (black) and empty (white) squares represent AORs, and the vertical bars represent 95% CIs

The results of the gender-stratified analyses are shown in Table 3. Figure 3 shows an AOR with a 95% CI for each health outcome by gender. Female telecommuters showed a higher risk of depression than daily commuters in both the unadjusted (OR = 1.53; 95% CI, 1.00-2.34) and fully adjusted models (AOR = 1.62; 95% CI, 1.04–2.53). However, this result was not observed in the analysis of male participants (unadjusted model: OR = 1.10; 95% CI, 0.73–1.67 and adjusted model 2: AOR = 1.11; 95% CI, 0.70–1.76). Another major gender difference was observed in the association between telecommuting and insomnia. Among male participants, a significant association between telecommuting and insomnia was observed in the unadjusted model (OR = 1.97; 95% CI, 1.05–3.70), although the association was no longer significant after adjustment for all potential covariates (AOR = 1.78; 95% CI, 0.89–3.56).

**Table 3:** Odds ratios with 95% CIs for health-related outcomes of telecommuters stratified by gender

	N (%)^a^		Unadjusted Model	Adjusted Model1^b^	Adjusted Model2^c^
Male					
Depression	94 (28.02)		1.10 (0.73–1.67)	1.13 (0.72–1.77)	1.11 (0.70–1.76)
Anxiety	47 (13.86)		2.88 (1.69–4.90)^*^	2.80 (1.60–4.89)^*^	2.93 (1.69–5.08)^*^
Insomnia	41 (12.18)		1.97 (1.05–3.70)^*^	1.77 (0.88–3.54)	1.78 (0.89–3.56)
Fatigue	94 (27.98)		1.67 (1.13–2.48)^*^	1.62 (1.08–2.45)^*^	1.61 (1.06–2.43)^*^
Musculoskeletal pain	142 (42.36)		2.16 (1.51–3.09)^*^	1.99 (1.34–2.95)^*^	1.92 (1.29–2.86)^*^
Headache, Eye strain	136 (40.57)		2.04 (1.44–2.90)^*^	1.88 (1.28–2.77)^*^	1.83 (1.24–2.69)^*^
Absenteeism	15 (4.43)		1.49 (0.72–3.05)	1.46 (0.69–3.07)	1.45 (0.68–3.09)
Presenteeism	49 (14.61)		1.74 (1.08–2.81)^*^	1.67 (1.03–2.72)^*^	1.66 (1.00-2.73)^*^
Female					
Depression	106 (34.70)		1.53 (1.00-2.34)^*^	1.52 (0.98–2.37)	1.62 (1.04–2.53)^*^
Anxiety	46 (14.74)		2.74 (1.69–4.44)^*^	2.51 (1.50–4.20)^*^	2.71 (1.61–4.57)^*^
Insomnia	52 (16.80)		2.24 (1.45–3.45)^*^	1.97 (1.22–3.18)^*^	2.07 (1.26–3.41)^*^
Fatigue	108 (34.52)		1.76 (1.16–2.66)^*^	1.82 (1.18–2.81)^*^	1.94 (1.25–2.99)^*^
Musculoskeletal pain	142 (45.45)		1.70 (1.20–2.42)^*^	1.61 (1.12–2.31)^*^	1.64 (1.13–2.38)^*^
Headache, Eye strain	134 (42.91)		2.02 (1.42–2.86)^*^	2.04 (1.41–2.96)^*^	2.08 (1.43–3.03)^*^
Absenteeism	21 (6.62)		1.37 (0.75–2.53)	1.29 (0.68–2.46)	1.29 (0.68–2.47)
Presenteeism	65 (20.97)		1.61 (1.08–2.39)^*^	1.59 (1.05–2.42)^*^	1.61 (1.05–2.48)^*^

CI: confidence interval

^a^Number and percentage of telecommuters affected in each outcome

^b^Adjusted for age, education, income, household size

^c^Adjusted for age, education, income, household size, working hour, shift work, occupation type

^*^P-value < 0.05

## Discussion

This study investigated the association between health-related outcomes and telecommuting by comparing comprehensive issues, including physical health, mental health, absenteeism, and presenteeism between telecommuters and daily commuters in South Korea. Six hundred fifty workers met the telecommuting definition; among them, 338 were males (312 females). Compared with daily commuters, telecommuters showed a higher prevalence of anxiety, insomnia, fatigue, musculoskeletal pain, headache/eye strain, and presenteeism. In addition, there were gender differences in the health status of telecommuters. For both depression and insomnia, a significant association between telecommuting was observed only in females. Overall, female workers seemed to be more susceptible to mental illness due to telecommuting.

Following the selection criteria of our study, 4.21% (n = 650) of all the participants (n = 15,451) were classified as telecommuters, which is a two-fold increase from the 5th KWCS (Table S1). Considering that the previous survey did not encompass the questionnaires on ICT device utilization, comparison between the two data should be performed with caution. The proportion of telecommuters in the working population of South Korea during the pandemic has been explored in prior studies using other secondary data. The 23rd Korean Labor and Income Panel Study identified the status of flexible work arrangements in March 2020 and showed 2.27% of paid workers worked from home [26]. Similarly, the South Korean Economically Active Population Survey held in August 2020 revealed an upsurge in telecommuters from 0.47% in 2019 to 2.49% in 2020 among paid workers [18].

Our findings are consistent with those of previous studies in which telecommuters showed worse health indicators than commuters. A cross-sectional study using data from the 2010, 2012, and 2013 American Time Use Survey assessed the subjective well-being of 3,962 wage workers and showed that telework was associated with higher psychological stress than office work, with coefficients of 0.298 (p-value of < 0.01) from fixed-effect regression models [27]. Another cross-sectional study investigated the effect of teleworking on physical discomfort among university faculty and staff who were forced to transition into teleworking during the COVID-19 pandemic. A total of 131 university members (86%) complained of new or worsening physical discomfort after telecommuting, while 7% reported improvements in their existing problems [28]. One multilevel regression from 25,465 workers in the 6th EWCS data showed that teleworking several times a week or daily resulted in a higher probability (11%) of experiencing presenteeism at least once per year with never teleworking as a reference [29]. However, the results of other studies differ from those of the present study. Henke et al. (2016) analyzed self-reported data from 3,703 financial workers. The results showed that telecommuters (≥ 73 h/month) had a lower overall health risk score than non-telecommuters (coefficient, -1.233; p-value < 0.05) [24]. A retrospective case-control study using screening data from 1,978 South Korean workers during the COVID-19 pandemic suggested that certain workplace interventions, including telecommuting, led to a significant decrease in depression and anxiety [30]. The former study differs from our research by analyzing only a specific occupation group, while the latter integrated paid leave and telecommuting into a single intervention. Such differences in study design would have led to opposite results.

To understand the link between telecommuting and its detrimental influence on health, differences in the working environment between remote and physical offices should be considered. A major concern of telecommuting is the blurring of boundaries between work and private life. Telecommuters are more likely to engage in less structured, longer working hours and have non-regular work schedules [7, 31, 32]. The negative impact of long working hours, such as chronic fatigue and physical/mental health problems, is well established [33]. These intense and extended hours at remote workstations have been associated with increased physical discomfort, musculoskeletal pain, burnout, and eyestrain [34]. A high prevalence of insomnia was found among employees with non-regular working patterns [35]. Telecommuters are also at greater risk of developing poor dietary habits and scarce exercise [36, 37]. Such unhealthy lifestyles can, in turn, cause workers’ fatigue and negatively affect their mental well-being [36, 38]. Another proposed risk factor for telecommuters is inappropriate ergonomic environment. Frequent use of non-office equipment, including chairs without armrests or laptops with no external monitor, exacerbates neck, shoulder, and lower back pain among telecommuters [39, 40]. Additionally, during the transition to remote work, telecommuters may lose resources at work, such as coworkers’ support, resulting in social isolation [41]. Many studies have suggested that social isolation and lack of support from coworkers lead to health impairments and are predictive of depressive disorders and burnout [42, 43]. The role of social support in moderating psychosocial stress responses is well known [44]. Regardless of widespread social distancing due to COVID-19, social isolation among telecommuters has consistently been observed [13, 45]. Vander Elst et al. (2017) demonstrated that the extent of telecommuting is negatively related to well-being because of the lack of social support from colleagues [46]. More importantly, during the global COVID-19 pandemic, the number of companies and governments implementing social distancing has surged, requiring employees to telecommute. Given that workers who engaged in involuntary teleworking reported greater emotional exhaustion than their voluntary counterparts, this sudden and unplanned shift to remote work may exacerbate the negative impact of telecommuting on their mental and physical health statuses [47]. The higher risk of presenteeism among telecommuters is partly attributable to their attitudes toward working from home. Notably, telecommuters may perceive their condition as a privilege to work comfortably at home and have the motivation to work incessantly in order not to miss the opportunity even if their health is compromised [48].

Interestingly, we found that female workers were more likely to have telecommuting-related health problems than male workers, indicating a gender difference in the effect of telecommuting on health-related outcomes. Similar findings were also reported in two recent studies [15, 49]. Giménez-Nadal et al. (2020) analyzed data from the American Time Use Survey and observed gender differences in the well-being of teleworkers, showing that male teleworkers had significantly lower levels of subjective stress, pain, and tiredness than commuters, while corresponding results were not found among female workers [15]. Graham et al. (2021) studied Australian telecommuters during the pandemic and found that females had higher levels of musculoskeletal discomfort/pain and psychosocial stress (OR = 2.06; 95% CI:1.38–3.08) than males [49]. The observed gender difference may be partially explained by the fact that teleworking has different connotations for males and females due to traditional gender roles.

In traditional households, females are responsible for domestic roles such as house chores and parenting, especially in East Asian countries [48, 50]. Excessive household chores are known to decrease work productivity, cause telecommuters to disengage from work, and increase job stress and sleep disturbances [13]. Although telecommuters spend less time commuting and gain more free time, females tend to invest extra time in household chores, unlike males, who are able to reinforce work-life balance with more leisure time [6, 51]. This may lead to a higher workload for female teleworkers, and the demands of work and family are combined, which become more harmful to their health. In addition, although it is unclear whether females are more vulnerable to social isolation caused by telecommuting, female workers reported feeling more loneliness and anxiety than males during the COVID-19 pandemic. [51, 52] A combination of disconnected relationships from co-workers and restrictions on social activity may pose a threat to the mental health of female telecommuters.

To the best of our knowledge, this is the first study to describe multifaceted, increased health problems among telecommuters in relation to daily commuters during the COVID-19 pandemic. The strength of our study is based on a large-scale, representative sample of South Korean workers. By applying the definition of telecommuting as inclusion/exclusion criteria into the participant’s selection process, employees who work from home were screened out. Our study also included overall health issues, specifically mental/physical health conditions and absenteeism/presenteeism, and we considered the gender aspects of telecommuting-related health problems. However, this study has several limitations. Most importantly, this was a cross-sectional study with its inherent inability to certify a causal relationship between exposure and outcome. In other words, whether telecommuting itself causes physical or mental illness or whether unhealthy workers are more inclined to work from home is unclear. Second, there were a few defects in the setting of independent variables. Although telecommuters’ workplace covers all non-central office spaces where both employers and employees agree, the current survey only inquired about “home” as a telecommuting place. The classification criteria for distinguishing telecommuters from commuters were a consequence of the author’s discretion, but were not referred from previous studies. Moreover, as the questionnaire item regarding telecommuting status followed qualitative criteria, we could not evaluate the quantitative intensity of telecommuting. Third, due to a lack of survey items, other possible confounders, such as lifestyle information (i.e. drinking and smoking), personal attitudes toward work, residential environment, or past medical history were not measured. Finally, rather than documented medical insurance claims or clinical records, outcome variables were measured by responses to questionnaires; therefore, a recall bias cannot be ruled out.

## Conclusion

During the COVID-19 pandemic, white-collar and salaried telecommuters were more likely to experience health problems (i.e., anxiety, fatigue, musculoskeletal pain, and headache/eye strain) than daily commuters. The association varied by gender, with female teleworkers experiencing a greater likelihood of depression and insomnia. Although further cohort and intervention studies are required to investigate causality, our study provides evidence of various negative health effects associated with telecommuting. To protect and manage telecommuters’ health, relevant organizations should monitor remote workers and develop appropriate measures to promote their health.

## Electronic supplementary material 

Below is the link to the electronic supplementary material.

Supplementary Material 1: **Table S1** Proportion of telecommuters among white-collar paid workers in South Korea. Additional table was presented to compare between 5th and 6th KWCS data, clarifying increasing trend of the numer of telecommuters during the pandemic era.

## Data Availability

The data utilized for the analysis of this study is publicly disclosed and available from the Korean Occupational Safety and Health Research Institute online repository: https://oshri.kosha.or.kr/eoshri/resources/KWCSDownload.do.

## Abbreviations

ICT: information and communications technology
IT: information technology
KWCS: Korean Working Conditions Survey
EWCS: European Working Conditions Survey
WHO: World Health Organization
MISS: Minimum Insomnia Symptom Scale
KRW: Korean Won
N: number
OR: odds ratio
AOR: Adjusted odds ratio
CI: confidence interval
