# Supplementary material for: Telecommuting-related health outcomes during the COVID-19 pandemic in South Korea: a national population-based cross-sectional study
Source: BMC Public Health. 2023 Mar 23;23:549. doi: 10.1186/s12889-023-15271-0 (PMC10034878; doi:10.1186/s12889-023-15271-0)
Supplement: Supplementary file 1 — Supplementary Material 1: Table S1 Proportion of telecommuters among white-collar paid workers in South Korea. Additional table was presented to compare between 5th and 6th KWCS data, clarifying increasing trend of the numer of telecommuters during the pandemic era. [file 12889_2023_15271_MOESM1_ESM.docx]

| **Table S1** Proportion of telecommuters among white-collar paid workers in South Korea | | | | | |
| --- | --- | --- | --- | --- | --- |
|  | Telecommuter | | Daily commuter |  |  |
| Survey period | Male | Female |  |  |  |
| 6th KWCS^a^ (October 2020 ~ April 2021) | 338 (2.19%) | 312 (2.02%) | 14,801 (95.79%) |  |  |
| 5th KWCS^b^ (July 2017 ~ November 2017) | 185 (1.04%) | 190 (1.06%) | 17,484 (97.90%) |  |  |
| a: Telecommuter defined as white-collar, paid worker using ICT devices who works from home  b: Telecommuter defined as white-collar, paid worker who works from home | | | | |  |
